# TiSe_2_-mediated sonodynamic and checkpoint blockade combined immunotherapy in hypoxic pancreatic cancer

**DOI:** 10.1186/s12951-022-01659-4

**Published:** 2022-10-15

**Authors:** Libin Chen, Wang Xue, Jing Cao, Shengmin Zhang, Yiqing Zeng, Ling Ma, Xuechen Qian, Qing Wen, Yurong Hong, Zhan Shi, Youfeng Xu

**Affiliations:** 1 Department of Ultrasound in Medicine, Ningbo First Hospital, Ningbo, 315010 People’s Republic of China; 2 Tongji University School of Medicine, Shanghai, 200072 People’s Republic of China; 3 Department of Ultrasound in Medicine, The Second Affiliated Hospital of Zhejiang University School of Medicine, No.88 Jiefang Road, Shangcheng District, Hangzhou, 310009 People’s Republic of China; 4 Research Center of Ultrasound in Medicine and Biomedical Engineering, The Second Affiliated Hospital of Zhejiang University School of Medicine, Zhejiang University, Hangzhou, 310009 China; 5 Department of Ultrasound in Medicine, Ningbo Ninth Hospital, Ningbo, 315032 People’s Republic of China

**Keywords:** Sonodynamic therapy, DC maturation, Anti-PD-1, TiSe_2_, Pancreatic cancer

## Abstract

**Background:**

Pancreatic cancer remains among the most prevalent and aggressive forms of cancer. While immunotherapeutic treatment strategies have shown some promise in affected patients, the benefits of these interventions have been limited by insufficient tumor infiltration by activated T cells.

**Results:**

Here, Titanium diselenide (TiSe_2_) nanosheets were synthesized with good stability. When exposed to ultrasound (US), the TiSe_2_ nanosheets served as a reliable nano-sensitizer capable of inducing large amounts of reactive oxygen species (ROS) mediating sonodynamic therapy (SDT) under hypoxic and normoxic conditions. The tumor-released TAAs induced by TiSe_2_ nanosheet-mediated SDT promoted immunogenic cell death (ICD) conducive to the maturation of dendritic cells (DCs), and cytokine secretion and the subsequent activation and infiltration of T cells into the tumor. Combining TiSe_2_-mediated SDT with anti-PD-1 immune checkpoint blockade treatment led to the efficient suppression of the growth of both primary tumor and distant tumor, while simultaneously preventing lung metastasis. These improved immunotherapeutic and anti-metastatic outcomes were associated with activated systematic antitumor immune responses, including the higher levels of DC maturation and cytokine secretion, the increased levels of CD8^+^ T cells and the decreased levels of T_reg_ cells infiltrated in tumors.

**Conclusion:**

TiSe_2_ can be used as a sonosensitizer with good efficacy and high safety to mediate efficient SDT. The combination treatment strategy comprised of TiSe_2_-mediated SDT and PD-1 blockade activate anti-tumor immune responses effectively thorough inducing ICD, resulting in the inhibition the growth and metastasis of tumor. The combination therapy holds promise as a novel immunotherapy-based intervention strategy for pancreatic cancer patients.

**Supplementary Information:**

The online version contains supplementary material available at 10.1186/s12951-022-01659-4.

## Background

Pancreatic cancer is an increasingly prevalent cause of cancer-related mortality, with a 5-year survival rate of under 10% in the USA [1, 2]. These poor prognostic outcomes are attributable to the fact that upwards of 90% of patients present with metastatic disease or are otherwise ineligible for tumor resection, although even for patients that do undergo tumor resection, the 5-year survival rate is just 20% [3–5].

Significant clinical success has been achieved in recent years through the development of immune checkpoint blockade (ICB) therapies based on the use of monoclonal antibodies specific for programmed cell death protein 1 (PD-1)/ programmed cell death ligand 1 (PD-L1) and cytotoxic T lymphocyte antigen 4 (CTLA-4) that can support the eradication of certain tumors and can protect against metastasis or recurrence via augmenting antitumor immune responses [6–9]. These ICB approaches, however, fail to confer significant benefit to most patients diagnosed with pancreatic cancer, exhibiting low response rates that limit their clinical utility despite initially promising Phase I trial results [10–12]. This is at least partially attributable to the fact that pancreatic tumors typically exhibit a tumor microenvironment (TME) that is considered to be immunologically “cold” owing to limited activation marker expression and low levels of tumor infiltration [13, 14]. As such, the combination of ICB and other therapeutic modalities capable of promoting intratumoral T cell infiltration and activation represents a promising approach to treating pancreatic cancer and preventing metastatic progression or tumor recurrence [15–17].

Ultrasound (US) exposure, which entails the generation of a mechanical wave outside the human auditory range with a frequency of > 20 kHz, represents a highly portable, noninvasive, and inexpensive therapeutic tool that can achieve excellent tissue penetration [18–20]. In addition to their widespread use in the context of medical imaging, US-based platforms and tools have also been studied in the context of cancer treatment [19, 21–23]. US-triggered sonodynamic therapy (SDT) can kill tumor cells by inducing the generation of high levels of singlet oxygen (^1^O_2_) and other reactive oxygen species (ROS), thereby initiating necrotic or apoptotic immunogenic cell death (ICD) [24, 25]. ICD induction can result in the release of tumor-associated antigens (TAAs) and damage-associated molecular patterns (DAMPs) from tumor cells, driving the activation of dendritic cells (DCs) and other antigen-presenting cell (APC) populations [26, 27]. US can penetrate tissue to a depth of 10 cm, thus offering a greater penetration depth than that which can be achieved with near-infrared (NIR) light such that US-triggered SDT represents a more viable clinical strategy than the related photothermal therapy (PTT) approach triggered by NIR irradiation [28, 29]. Titanium diselenide (TiSe_2_) is a two-dimensional transition metal dichalcogenide (TMDC) that exhibits promising photoresponsivity such that it has been leveraged in photodynamic therapy (PDT) treatment applications [30]. However, whether two-dimensional TiSe_2_-based nanomaterials can similarly be used to facilitate sonosensitization remains to be established.

**Scheme 1:**
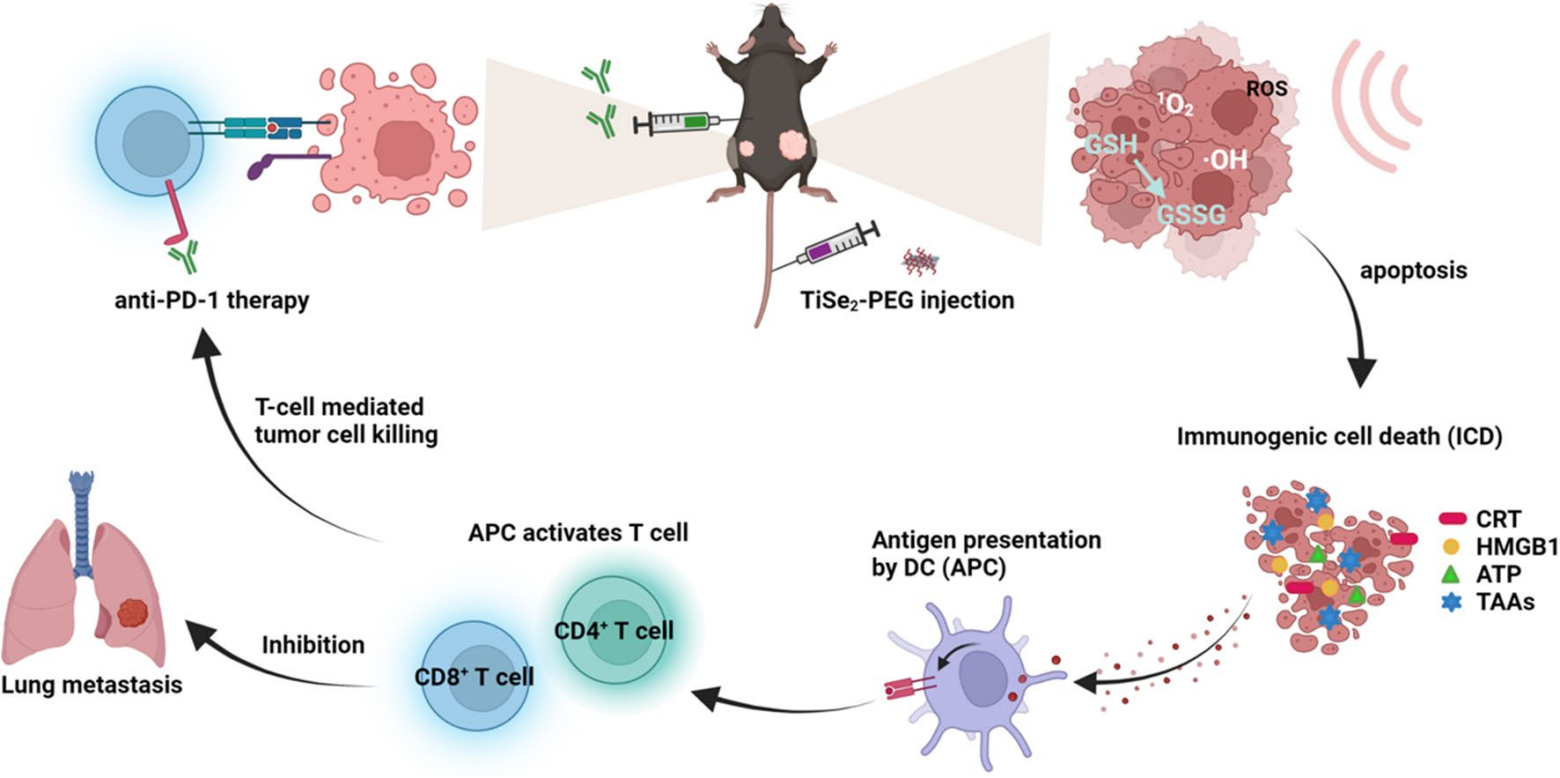
A schematic illustration of the combined application of TiSe_2_-mediated SDT and anti-PD-1 immune checkpoint blockade for the treatment of pancreatic cancer. SDT, sonodynamic therapy; PD-1, programmed cell death protein 1; ICD, immunogenic cell death; DC, dendritic cell; APC, antigen-presenting cell; CRT, calreticulin; HMGB1, high mobility group box 1; ATP, adenosine triphosphate; TAAs, tumor-associated antigens

Given the promise of SDT as a therapeutic modality and its ability to trigger the induction of an immune response, the value of combining TiSe_2_-mediated SDT and PD-1 blockade when treating pancreatic cancer in vitro and in vivo was herein evaluated. When subjected to US irradiation, TiSe_2_ nanosheets were found to generate high levels of cell death in normoxic and hypoxic settings. The TAAs released from primary tumors exposed to TiSe_2_ nanosheet-mediated SDT were sufficient to promote DC maturation and cytokine secretion, thereby effectively engaging a robust antitumor immune response. When this SDT approach was applied in combination with anti-PD-1 checkpoint blockade therapy, systemic antitumor immunity was successfully induced in the mouse model system, as evidenced by increases in intratumoral CD8^+^ T cell infiltration and decreases of T_reg_ cells infiltration. This combination approach was sufficient to both inhibit primary tumor growth and slow metastatic pancreatic tumor progression (Scheme 1). Accordingly, combining SDT and immune checkpoint blockade strategies represents a promising approach to treating pancreatic cancer.

## Experimental section

### TiSe_2_ nanosheet synthesis

Growth of TiSe_2_ Crystals: To produce TiSe_2_ crystals, bulk TiSe_2_ was prepared via a CVT approach. Briefly, ball-milling was used to combine Se powder (S105195, Aladdin), Ti powder (T109127, Aladdin), and I_2_ powder (I116353, Aladdin) for 2 h under vacuum conditions, after which the mixed powder was separated from these balls and allowed to fully dry prior to transfer into quartz tubes. After cooling to room temperature for 30 min, tubes were heated to 700 °C within 70 min, kept at 700 °C for 30 min, cooled to 200 °C within 6 h, and then cooled to room temperature following a 30 min incubation, thereby producing bulk TiSe_2_ crystals.

Synthesis of TiSe_2_ nanosheets: A liquid exfoliation approach was used to synthesize TiSe_2_ nanosheets via ultrasound probe sonication with subsequent bath sonication of ground bulk TiSe_2_ powder. Briefly, 30 mg of the bulk TiSe_2_ crystals prepared above were combined with 25 mL of DMF in a 50 mL tube, followed by sonication (1200 W, 10 h, 6–8 °C) with an ultrasonic frequency of 25 kHz, operating the ultrasound probe for 2 s at 4 s intervals. The dispersed mixture was then subject to continuous sonication in an ultrasonic bath (300 W, 10 h, < 15 °C). The resultant mixture was centrifuged at 5,000 rpm for 10 min, after which the TiSe_2_ nanosheet-containing supernatant fraction was gently poured into a 50 mL conical tube followed by an additional round of centrifugation at 14,000 rpm for 10 min. Precipitates were then rinsed using ethanol and water, followed by resuspension in an appropriate aqueous medium.

Modification with PEG: To improve colloidal stability, PEG modification was achieved by suspending 1 mg of TiSe_2_ nanosheets in 5 mL of H_2_O and then adding 10 mg of CH_3_-PEG-SH. Following ultrasonication for 15 min, samples were stirred for 6 h, after which free PEG was removed by centrifuging samples at 12,000 rpm for 10 min and washing with water three times. The resultant PEG-modified TiSe_2_ nanosheets were suspended in an appropriate aqueous medium.

### Nanosheet characterization

Transmission electron microscopy (TEM) and high-resolution TEM (HR-TEM) were used to assess the structural characteristics of TiSe_2_ nanosheets. X-ray diffractometry (XRD) was used to assess the structural performance of TiSe_2_ nanosheets. The elemental composition and valence states of TiSe_2_ nanosheets were verified via X-ray photoelectron spectroscopy (XPS). The heights of four typical TiSe_2_ nanosheets were examined via atomic force microscopy (AFM). The dynamic light scattering (DLS) characteristics and zeta potential TiSe_2_ nanosheets were assessed with a particle analyzer (Nano-ZS, Malvern, England) at room temperature.

### ROS generation analyses

The production of ^1^O_2_ radicals was monitored using the singlet oxygen sensor green (SOSG) probe (S36002, Thermo Fisher Scientific, MA, USA), while an aminophenyl fluorescein (APF) assay (A4108, Sigma-Aldrich, USA) was employed to monitor the generation of ·OH radicals. For these analyses, PBS was used to resuspend TiSe_2_ nanosheets at a range of concentrations (0, 10, 20, 30, 40, or 50 µg/mL), after which they were subjected to US irradiation (0.5 W/cm^2^, duty rate 50%, 1 min, 1 MHz, Mettler Sonicator 740). Alternatively, a fixed TiSe_2_ nanosheet concentration (25 µg/mL) was subjected to US irradiation for 0, 10, 30, 60, 90, 120, or 150 s (0.5 W/cm^2^, duty rate 50%, 1 MHz). Fluorescence values in these samples were then detected via multiscan spectrum (Tecan, Swiss).

### In vitro cell-level analyses of ^1^O_2_ production

The ability of TiSe_2_ nanosheets to promote in vitro cellular ^1^O_2_ generation upon US irradiation was assessed using Panc02 cells divided into four groups: control untreated cells, cells treated with TiSe2 nanosheets, cells treated with US alone, or cells treated with TiSe_2_ nanosheets + US. The TiSe_2_ treatment dose in all appropriate groups was fixed at 25 μg/mL, and cells were treated for 8 h at 37℃, after which culture media was replaced with DCFH-DA (S0033, Beyotime Biotechnology), and cells were subjected to US irradiation (1.0 MHz, 0.5 W/cm^2^, 50% duty cycle, 1 min). Following an additional 30 min incubation, cells were rinsed thrice in PBS and examined via confocal laser scanning microscopy (CLSM).

### Cell surface calreticulin (CRT) measurement

Panc02 cells were added to 35 mm confocal dishes (1 × 10^5^/well) and cultured for 12 h at 37 °C. Cells were then treated for 8 h with either PBS or TiSe_2_ nanosheets (25 µg/mL), after which they either were or were not subjected to US irradiation (1.0 MHz, 0.5 W/cm^2^, 50% duty cycle, 1 min). Following an additional 24 h incubation, cells were fixed for 15 min with 4% paraformaldehyde (PFA) in PBS at room temperature, rinsed three times using PBS, and stained overnight with anti-CRT (Abcam, Cambridge, UK, ab91654) at 4 °C. Following three additional washes with PBS, cells with incubated with AF594-conjugated secondary antibodies and Dio-488 (to stain the cell surface) for 1 h at room temperature. Nuclei were then stained using DAPI, and CRT exposure was visualized by CLSM.

Cells were additionally harvested for flow cytometric analyses of CRT surface exposure. Briefly, treated cells were collected, rinsed three times, and incubated for 1 h on ice with primary anti-CRT in 2% FBS. Cells were then rinsed with PBS and probed with an AF594-conjugated secondary antibody in 2% FBS for 1 h. Samples were then analyzed via flow cytometry to detect cell surface CRT exposure.

### High mobility group box 1 (HMGB1) release assay

Cells were added to 35 mm confocal dishes (1 × 10^5^/dish) and incubated for 12 h, after which they were treated using the same conditions detailed in the CRT experiment above. Cells were then washed thrice with PBS, fixed for 10 min with 4% paraformaldehyde, permeabilized for 10 min using 0.1% Triton X-100, and rinsed three times using cold HBSS. Cells were then blocked for 30 min in 10% FBS, stained for 1 h with anti-HMGB1 (ab18256, Abcam, Cambridge, UK), washed again, incubated for 1 h with AF488-conjugated secondary antibodies, and stained with DAPI. CLSM was then used to detect HMGB1 release.

The release of HMGB1 was additionally detected by Western immunoblotting. Briefly, after the treatments described above, supernatants were harvested, centrifuged to remove dead cells, and subjected to ultrafiltration to concentrate them to a final volume of 100 µL. Adherent cells were lysed using RIPA buffer and centrifuged, after which the supernatants and cell lysate samples were separated via SDS-PAGE and the resultant blots were probed with antibodies specific for HMGB1 and tubulin. Protein bands were detected using an enhanced chemiluminescence (ECL) approach (Millipore, Darmstadt, Germany).

### Adenosine triphosphate (ATP) secretion assay

Panc02 cells were added to 48-well plates (1 × 10^5^/well) and cultured for 12 h, after which they were treated using the same protocols employed for the CRT detection assays. Supernatants were then harvested, and dead cells were removed via centrifugation. The ATP assay (Beyotime Biotechnology Co., Shanghai, China, S0027) was then performed based on the manufacture’s instruction using 25 µL aliquots from each sample.

### In vitro analyses of the biocompatibility of TiSe_2_ nanosheets

A Cell Counting Kit-8 (CCK-8) assay (MCE, USA) was used to analyze the biocompatibility of synthesized TiSe_2_ nanosheets. For this assay Panc02, BxPC-3, MDA-MB-468, hTERT-HPNE or HUVEC cells were seeded in 96-well plates (5000/well) and incubated overnight. Media was then replaced with DMEM/RPMI-1640 containing various concentrations of TiSe_2_. Cells were incubated for 24 h, and then CCK-8 solution was added to each well, with absorbance then being measured at 450 nm in each well using a microplate reader after 1 h. SDT efficiency was additionally analyzed by adding Panc02, hTERT-HPNE or HUVEC cells to 96-well plates in a 100 µL volume and incubating them overnight, followed by an 8 h incubation with TiSe_2_ nanosheets (25 µg/mL). Cells were then exposed to US irradiation (0.5 W/cm^2^, 1 MHz, 50% duty cycle, 1 min), followed by an additional 16 h incubation. All treatment steps were performed under hypoxic and normoxic conditions. CCK-8 assays were then used to quantify cellular viability as above.

### In vivo biodistribution analyses of TiSe_2_ nanosheets

Panc02 cells (5 × 10^6^) were subcutaneously implanted in the right flank of mice to establish a murine tumor model. When the tumors were 50–100 mm^3^ in size, TiSe_2_ nanosheets (5 mg/kg) were administered intravenously to these Panc02 tumor-bearing mice. After 8 h, the tumors and major organs (heart, liver, spleen, lung, and kidney) were excised and subsequently homogenized to measure the Ti content by inductively plasma mass spectrometry (ICP-OES).

### In vivo analyses of the immunostimulatory effects of TiSe_2_-mediated SDT in tumor-bearing mice

C57BL/6 mice (males, 5–6 weeks old) were purchased from Bikai Biological Technology Co.Ltd and used for in vivo experiments performed according to protocols that had been approved by the Animal Laboratory of the Second Affiliated Hospital of Zhejiang University (permit NO. 2021–109). For initial experiments, mice were randomized into the following treatment groups (n = 5/ group): (1) control, (2) TiSe_2_, (3) US only, (4) TiSe_2_ + US. To establish a syngeneic murine tumor model, mice were subcutaneously implanted in the right flank with Panc02 cells (5 × 10^6^). Once tumors were 50–100 mm^3^ in size, the experimental treatments were initiated. SDT treatment was performed in appropriate mice on days 0, 1, and 2 of the treatment period. Animals in the control and US groups received intravenous (i.v.) injections of PBS, whereas mice in the other groups received an i.v. infusion of TiSe_2_ nanosheets (5 mg/kg). Mice in appropriate groups underwent US irradiation (1.0 MHz, 0.5 W/cm^2^, 50% duty cycle, 5 min) at 8 h post-injection. On day 4 of the treatment period, tumor draining lymph nodes were harvested from these mice for flow cytometry analysis of DC activation. Cells were stained with the Fixable Viability Kit-Zombie Red reagent (Biolegend, Catalog: 423,110), anti-CD11c BV421 (Biolegend, Catalog: 117,329), anti-CD80 PE (Biolegend, Catalog: 104,733) and anti-CD86 BV785 (Biolegend, Catalog: 105,043). Tumors were additionally collected from these mice for TUNEL and H&E staining, while blood samples were used for ELISA-based analyses of proinflammatory cytokines including TNF-α, IL-12 p40, and IL-6.

### Analysis of the effects of combination SDT and PD-1 blockade on distant tumor growth

A primary tumor model was established by subcutaneously implanting Panc02 cells (5 × 10^6^) in the right flank of each mouse. After 6 days, mice were then randomized into PBS, anti-PD-1, TiSe_2_ + US, and TiSe_2_ + US + anti-PD-1 treatment groups (n = 5/group). A second tumor was then established to simulate distant tumor growth by subcutaneously implanting Panc02 cells in the left flank. Animals were then intravenously injected with the same therapeutic regimens detailed above on days 0, 2, and 4. US irradiation was performed using the same parameters as above 12 h post-injection. Mice in appropriate treatment groups were injected with anti-PD-1 (75 μg/mouse) on days 1, 3, and 5. Tumor volume was calculated as follows: (width^2^ × length)/2. After experimental completion, mice were euthanized, and tumors were collected, weighed, and imaged.

A murine model of lung metastasis was established by administering 1 × 10^5^ Panc02 cells via the tail vein 6 days following initial tumor inoculation performed as above. Animals were then subjected to SDT and anti-PD-1 treatment as above. Primary tumors were excised surgically on day 4. Upon experimental completion, lungs from these animals were fixed with Bouin's solution. Micrometastases from five lobes of the lung were subject to pathological analyses. Mice bearing tumors > 1000 mm^3^ in size were euthanized in accordance with standard animal protocols.

### Exploration of the in vivo mechanisms underlying combined treatment efficacy

A systematic analysis of the in vivo induction of antitumor immune responses was performed by harvesting tumors from mice at appropriate time points and preparing a single-cell suspension by digesting these tumors for 2 h at 37 °C using DNAse and Collagenase IV in PBS. These cells were then stained using the Fixable Viability Kit-Zombie Red (Biolegend, Catalog: 423,110) and with the following fluorochrome-conjugated antibodies: CD45-BV605 (Biolegend, Catalog: 103,139), CD3-PerCP-Cy5.5 (Biolegend, Catalog: 100,218), CD4-FITC (Biolegend, Catalog: 100,509), CD8a-BV510 (Biolegend, Catalog: 100,751), CD25-APC (Biolegend, Catalog: 102,012) and Foxp3-PE (Biolegend, Catalog: 320,008). After labeling, cells were analyzed via flow cytometry.

### Statistical analysis

All experiments were repeated with a minimum of three independent measurements. Data are reported as means ± standard deviation (SD), and were analyzed using SPSS v 25.0.

## Results and discussion

### TiSe_2_ nanosheet synthesis and characterization

**Fig. 1:**
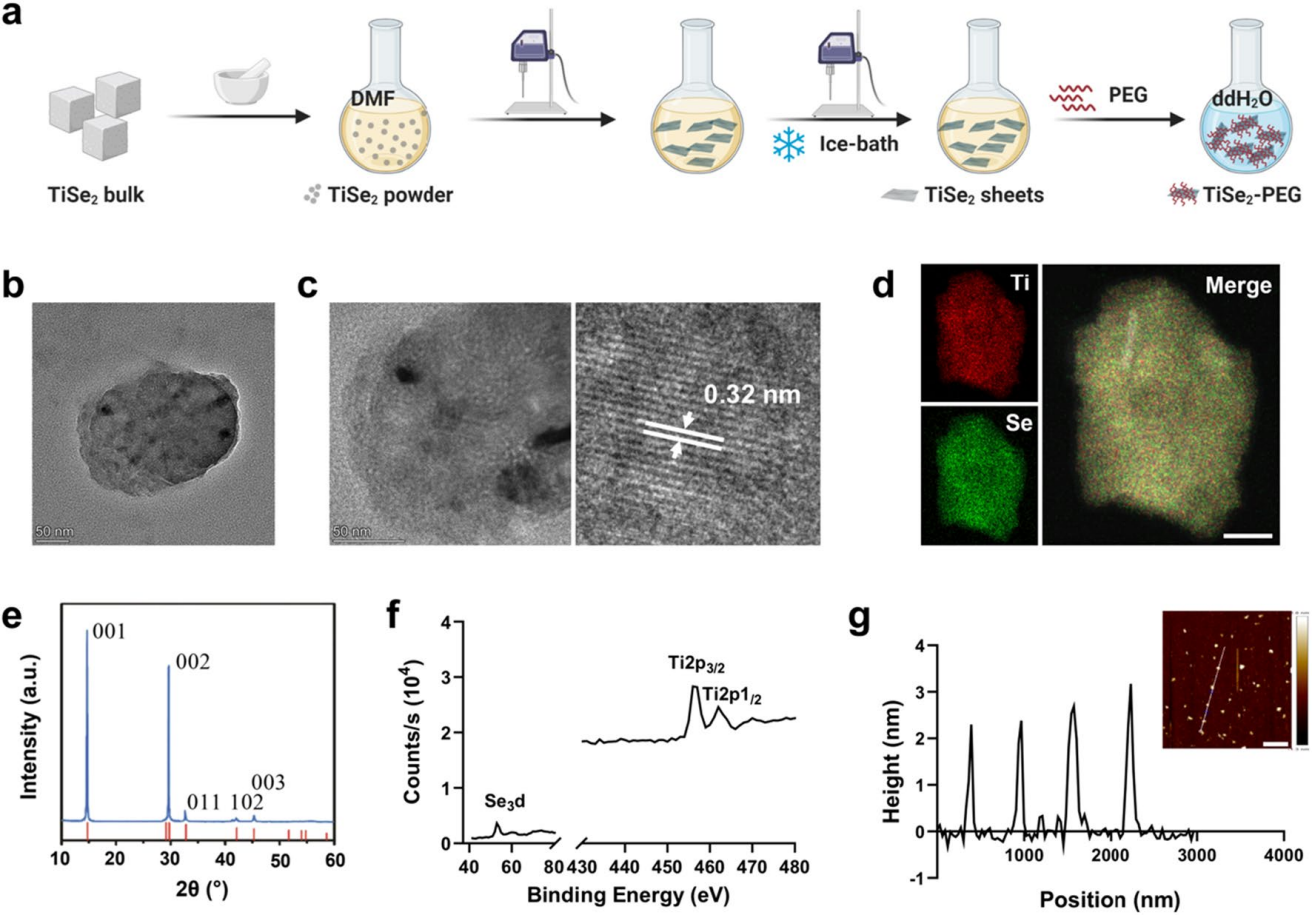
TiSe_2_ nanosheet structural analyses. **a** The approach used to synthesize TiSe_2_ nanosheets. TEM images (**b**), high-resolution TEM images (**c**), STEM-EDS maps (**d**), XRD patterns (**e**), XPS spectra (**f**), and AFM images and height profiles (**g**) for TiSe_2_ nanosheets

A general overview of the approach employed to synthesize TiSe_2_ nanosheets for use as sonosensitizers is summarized in Fig. 1a. Morphological characterization of the prepared nanosheets was performed by TEM and HR-TEM. As expected, these nanosheets exhibited sheet-like characteristics (Fig. 1b), and HR-TWM revealed 0.32 nm lattice fringes and multiple polycrystalline grains that were randomly oriented and amorphous in nature, with clear grain boundaries and apparent dislocation (Fig. 1c). The DLS indicated that the average hydrodynamic diameters of the TiSe_2_ nanosheets remained stable without obvious size variation for 7 days when incubated with PBS or FBS-containing DMEM (Additional file 1: Fig. S1). The STEM-EDS confirmed that both Se and Ti were present on the surface of the synthesized TiSe_2_ nanosheets (Fig. 1d), while the XRD-based characterization revealed five total diffraction peaks including three indexed to the (001), (002), and (003) facets, indicating the presence of the hexagonal TiSe_2_ crystal structures (Fig. 1e). The XPS-based analyses of the chemical composition of these nanosheets were also performed (Fig. 1f), revealing Se_3_d at 54.0 eV, Ti2p_3/2_ at 470.0 eV, and Ti2p_1/2_ at 462.0 eV. The AFM was used to assess the heights of four typical TiSe_2_ nanosheets, with an average measured height of 2.63 ± 0.39 nm (Fig. 1g). Together, these results confirmed the successful synthesis of TiSe_2_ nanosheets.

### Analyses of in vitro nanosheet-mediated ROS production

**Fig. 2:**
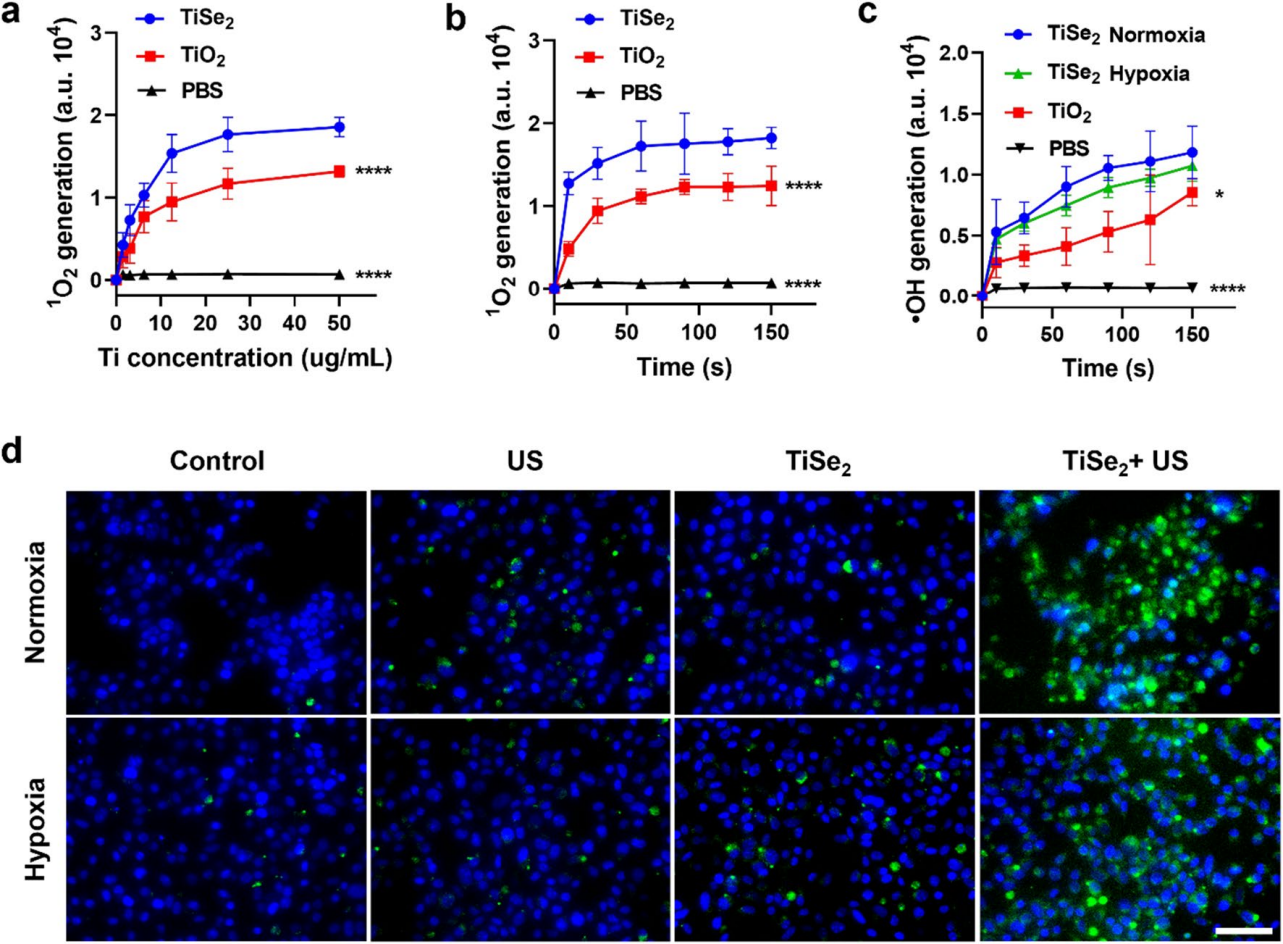
Analyses of in vitro ROS production. Cells were monitored for the dose-dependent (**a**) and time-dependent generation of ^1^O_2_ (**b**), as well as the time-dependent generation of ·OH generation (**c**), and the intracellular production of ROS within Panc02 cells as detected with DCFH-DA (**d**). US, ultrasound. Scale bar: 50 μm. Data are means ± SD (n = 3), and were compared via one-way ANOVAs with Dunnett’s post hoc test. *P < 0.05, ****P < 0.0001

The ability of these TiSe_2_ nanosheets to facilitate ROS generation was next assessed. When utilizing a SOSG probe to detect ^1^O_2_ generation ^31^, the ^1^O_2_ signal was found to rise in a dose- and time-dependent manner (Fig. 2a–b), with higher ROS yields than those generated by pure TiO_2_ nanoparticles. The generation of •OH radicals under varying oxygen partial pressure levels was also assessed, revealing time-dependent increases in APF fluorescence without any significant differences between the hypoxic and normoxic treatment conditions (Fig. 2c), consistent with oxygen-independent •OH production. Overall, these results suggested that TiSe_2_ nanosheets hold promise as a sonosensitizer for use in the treatment of hypoxic solid tumors, including pancreatic tumors.

ROS levels within cells were additionally measured using the DCFH-DA probe. Following TiSe_2_ nanosheet treatment and US irradiation for 1 min, these cells exhibited a bright green fluorescence that was less pronounced in the PBS, US, or TiSe_2_ treatment groups (Fig. 2d). GSH depletion was also analyzed in vitro as a measure of changes in the regulation of the intracellular oxidation-redox balance, revealing an up to 15.6% depletion of GSH following TiSe_2_ nanosheet treatment and US irradiation (Additional file 1: Fig. S2). In contrast, negligible GSH depletion was observed in control solutions containing GSH solution alone or TiSe_2_ nanosheets not subjected to US irradiation.

### Evaluation of ICD and cytotoxicity

**Fig. 3:**
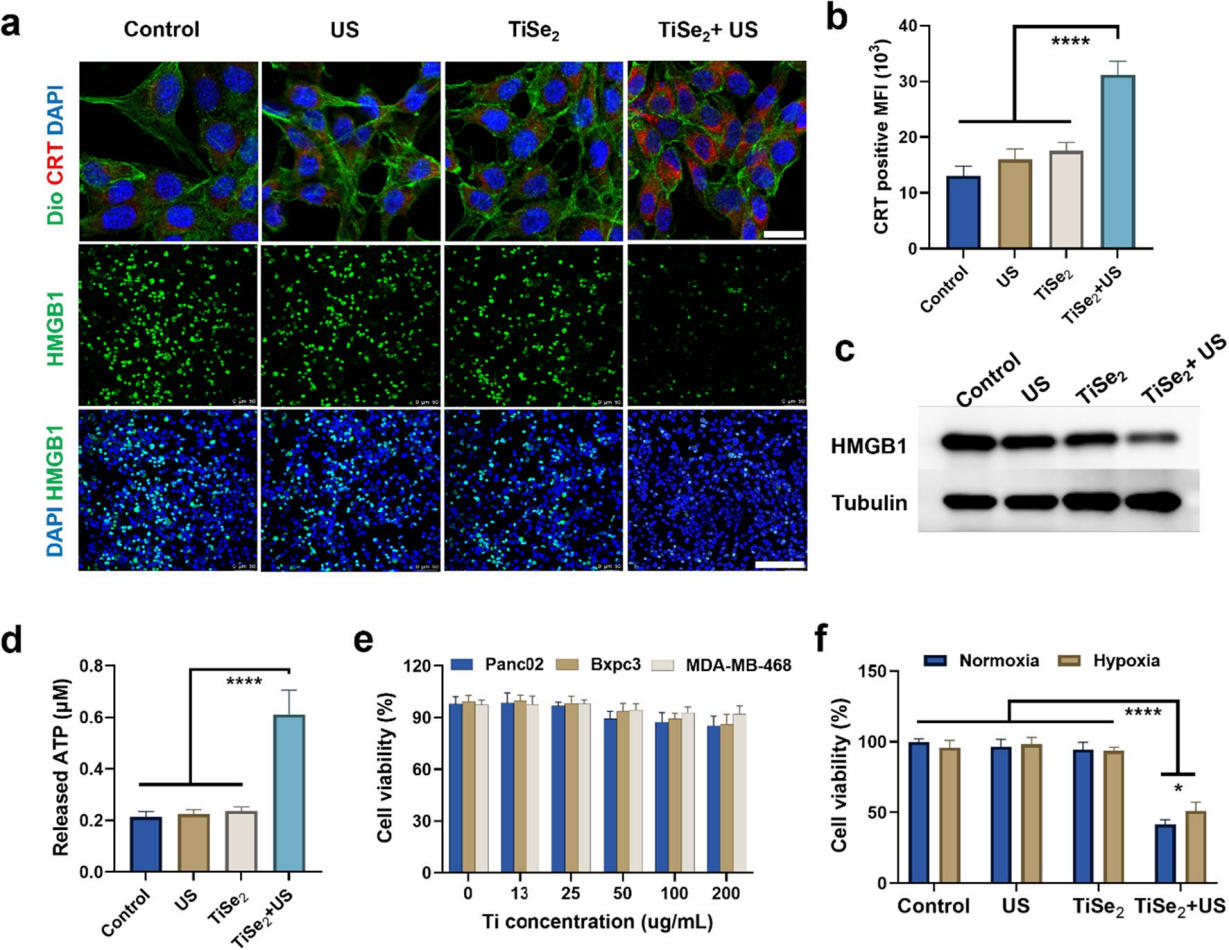
In vitro analyses of therapeutic toxicity and immunostimulatory activity. **A** Panc02 cells treated with the indicated conditions were assessed for CRT exposure and HMGB1 release via CLSM. Scale bars: 25 µm and 100 µm. **b** CRT exposure in the indicated treatment groups was examined via flow cytometry (n = 3). **c** HMGB1 release from Panc02 cells in the indicated groups was examined via Western blotting. **d** The release of ATP from Panc02 cells in the indicated treatment groups was assessed (n = 3). **e** TiSe_2_ cytotoxicity was quantified in Panc02, Bxpc-3, and MDA-MB-468 cells in the absence of US irradiation (n = 5). **f** CCK-8 analyses of Panc02 cells were conducted under hypoxic or normoxic conditions following treatments with the indicated formulations for 24 h (n = 5). CRT, calreticulin; HMGB1, high mobility group box 1; ATP, adenosine triphosphate; US, ultrasound. Data are means ± SD, and were compared via one-way ANOVAs with Bonferroni post hoc testing or Student’s t-tests. *P < 0.05, ****P < 0.0001

Next, CRT exposure, the release of HMGB1, and the secretion of ATP were measured to examine the ability of TiSe_2_ nanosheet treatment to induce ICD. Significant increases in CRT exposure and nuclear HMGB1 release were evident in Panc02 cells following TiSe_2_ nanosheet treatment and US irradiation (ICD) as detected via CLSM, consistent with ICD (Fig. 3a). Flow cytometry analyses further confirmed the increase of CRT exposure on cells in the TiSe_2_ + US irradiation group (Fig. 3b), whereas Western blotting revealed a significant decrease in intracellular HMGB1 expression in this group relative to the control, US, and TiSe_2_ treatment groups (Fig. 3c). Consistent with enhanced ROS-mediated damage to the mitochondria, higher levels of extracellular ATP were observed in samples from Panc02 cells subjected to TiSe_2_ + US treatment relative to other tested treatments (Fig. 3d). Overall, these findings supported ability of TiSe_2_-mediated SDT to induce the immunogenic death of pancreatic tumor cells in vitro*.*

A CCK-8 assay was next employed to gauge in vitro TiSe_2_ nanosheet cytotoxicity. In the absence of US irradiation, no significant decrease in cell survival was evident for any of the tested tumor cell lines (Panc02, MDA-MB-468, Bxpc-3), consistent with an absence of any intrinsic TiSe_2_ nanosheet-induced cell killing (Fig. 3e). Similarly, US irradiation alone failed to adversely affect the survival of Panc02 cells within the tested range (Additional file 1: Fig. S3). However, a marked drop in cell viability was observed under hypoxic and normoxic conditions when Panc02 cells were subjected to SDT through a combination of TiSe_2_ treatment and US irradiation (1.0 MHz, 0.5 W/cm^2^, 50% duty cycle, 1 min) (Fig. 3f), which had limited effect on the viability of normal cells (hTERT-HPNE and HUVEC) (Additional file 1: Fig. S4). These data thus further confirmed the ability of these TiSe_2_ nanosheets to serve as effective tools for use when treating hypoxic solid tumors with potential clinical application prospect.

### In vivo DC maturation and inflammatory cytokine production

**Fig. 4:**
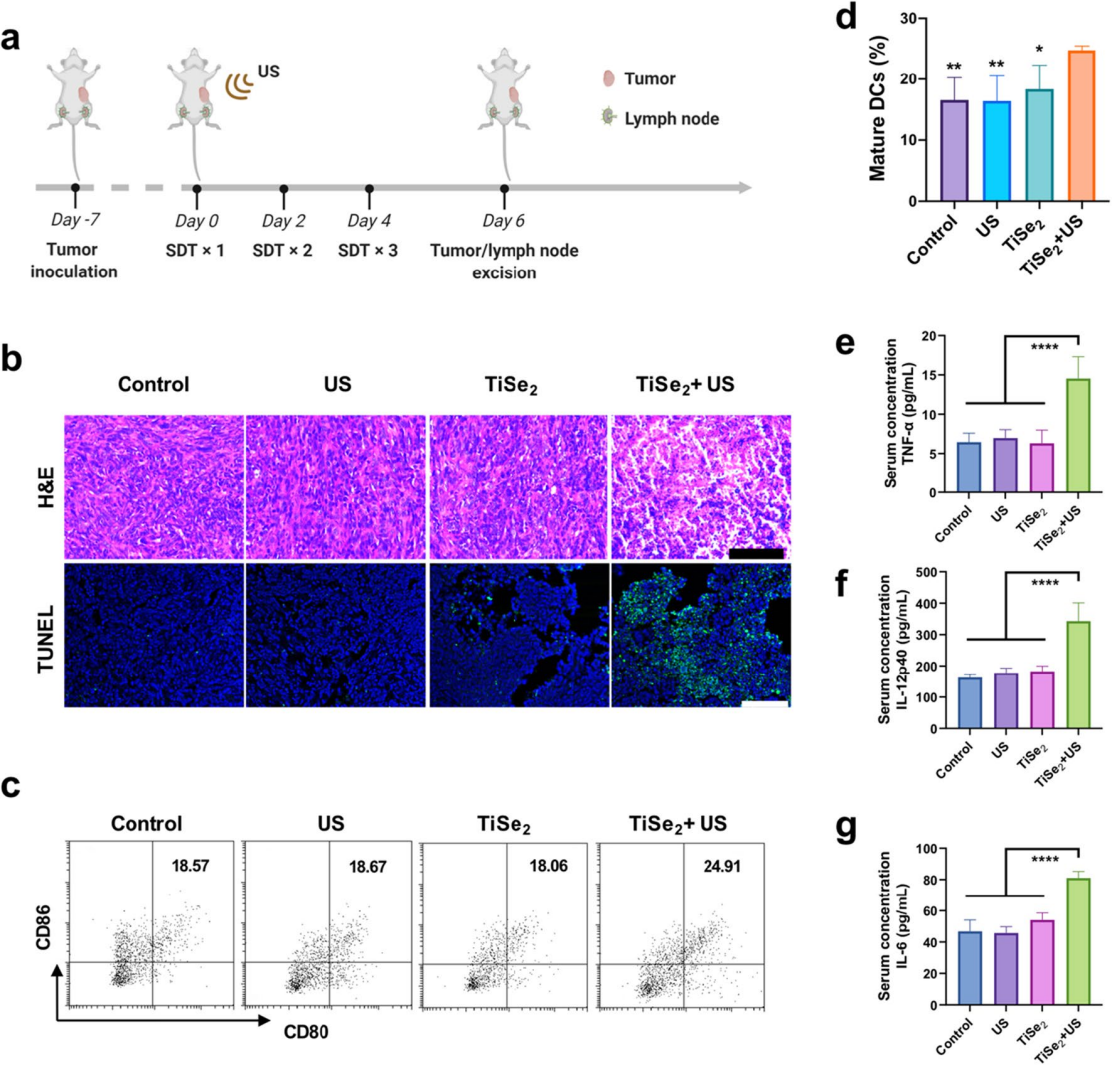
In vivo analyses of the ability of TiSe_2_-mediated SDT to promote the maturation of DCs and the production of proinflammatory cytokines. **a** Schematic overview of the experimental design employed to analyze in vivo immune responses to TiSe_2_-mediated SDT. **b** In vivo analyses of tumor cell apoptotic and necrotic cell death in response to TiSe_2_-mediated SDT treatment as detected via TUNEL and H&E staining. Scale bar: 100 µm. **c**, **d** The maturation of DCs was detected in tumor-draining lymph nodes from Panc02 tumor-bearing mice by flow cytometry following TiSe_2_-mediated SDT, with cells being stained with a Live/Dead stain and antibodies specific for CD11c, CD80, and CD86. **e**–**g** Murine serum TNF-α, IL-12p40, and IL-6 levels were quantified at 24 h post-TiSe_2_-mediated SDT treatment in the indicated groups. SDT, sonodynamic therapy; US, ultrasound; DCs, dendritic cells. Data are means ± SD (n = 5), and were compared via one-way ANOVAs with Bonferroni post hoc testing. *P < 0.05, ****P < 0.0001

Given the promising in vitro results shown above, the utility of TiSe_2_ nanosheets was next assessed in vivo in a syngeneic tumor-bearing mouse model system (Fig. 4a). Inductively plasma mass spectrometry (ICP-OES) were performed on the Panc02 tumor models to further validate the accumulation and biodistribution of TiSe2 nanosheets, which was showed to more sufficient accumulated in the liver, spleen and tumor tissues 8 h after the injection, suggesting that the nanosheets could actively accumulate into the tumor site through intravenously injection (Additional file 1: Fig. S5). H&E staining revealed clear evidence of nuclear fragmentation in tumor tissue sections from mice in the TiSe2 + US treatment group with a corresponding decrease in nuclear size, while TUNEL staining further indicated that TiSe2 + US treatment was associated with the significant induction of apoptotic tumor cell death (Fig. 4b). Flow cytometry analyses revealed that relative to other treatments, DC maturation was significantly enhanced by combined TiSe_2_ + US treatment (Fig. 4c-d), which also promoted proinflammatory cytokine production as measured by ELISA (Fig. 4e-g). These results suggested that such TiSe_2_ nanosheet-mediated SDT was conducive to the induction of a more robust antitumor immune response. Notably, higher levels of DC maturation were evident in the TiSe_2_ + US group (24.75%) relative to other groups. Following the SDT-induced death of tumor cells, the TAAs, DAMPs and other cellular debris shed by these cells could thus be transported to tumor-draining lymph nodes wherein they were able to promote the activation of DCs and other APCs in a manner conducive to the induction of cell-mediated immunity.

### Combined SDT and anti-PD-1 treatment suppresses distant tumor growth though the induction of systemic antitumor immunity

**Fig. 5:**
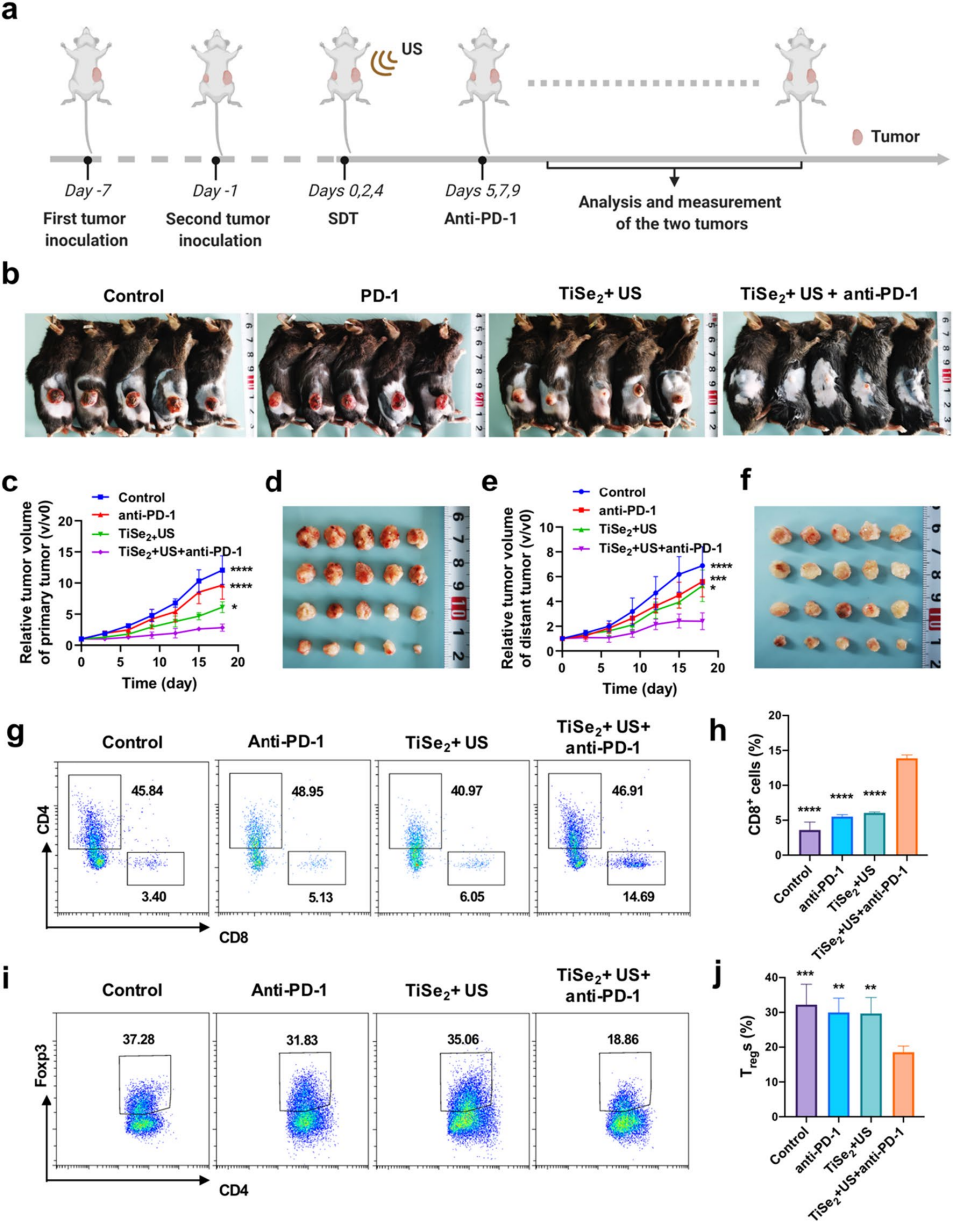
Analyses of the in vivo antitumor impact of combination TiSe_2_-mediated SDT plus anti-PD-1 treatment. **a** Schematic overview of the experimental approach used to evaluate the effects of TiSe_2_-mediated SDT and anti-PD-1 combination treatment in murine tumor models. **b** Representative images of mice at the experimental endpoint in the indicated treatment groups. **c**, **f** Primary tumor growth was monitored (**c**) and primary tumors were imaged in the indicated treatment groups, with distant tumor growth (**e**) and imaging (**f**) similarly being performed in these animals. **g**–**j** Representative flow cytometry plots and corresponding quantitative analyses of the infiltration of CD8^+^ T cells (CD45^+^CD8^+^) and CD4^+^ FoxP3^+^ T cells (CD45^+^CD4^+^FoxP3^+^) in distant tumors. SDT, sonodynamic therapy; US, ultrasound. Data are means ± SD (n = 5), and were compared via one-way ANOVAs with Dunnett’s post hoc test. **P < 0.01, ***P < 0.001 and ****P < 0.0001

To examine the ability of TiSe_2_-mediated SDT to synergize with anti-PD-1 blockade treatment as a means of achieving more robust antitumor immunity, mice bearing bilateral Panc02 tumors were next established (Fig. 5a). Briefly, an initial tumor was implanted in the left flank of each mouse, followed 6 days later by the implantation of another tumor in the right flank to establish an artificial model of distant metastasis. Following three rounds of combination TiSe_2_-mediated SDT + anti-PD-1 treatment, treatment outcomes were compared for against the primary and mimic distant metastatic tumors in these different groups were assessed (Fig. 5b–f). Anti-PD-1 treatment alone largely failed to impact primary or secondary tumor growth. TiSe_2_-mediated SDT alone was able to effectively suppress primary tumor growth (*P* < 0.0001) but failed to impact distant tumor growth. Critically, a combination of TiSe_2_-mediated SDT + anti-PD-1 treatment resulted in the near-total eradication of primary tumors together with significant suppression of the growth of distant secondary tumors (*P* < 0.0001). This combined treatment approach did not result in any weight loss (Additional file 1: Fig. S6) or temperature changes (Additional file 1: Fig. S7) in tumor-bearing mice, attesting to the excellent biosafety profile associated with the combination of SDT and immunotherapy.

To gain further insight regarding the mechanisms underlying the enhancement of antitumor immunity in mice subjected to TiSe_2_-mediated SDT and PD-1 blockade, immune cells were harvested from distant tumors in these animals on day 18 after the initial treatment. The abundance of CD8^+^ T cells in distant tumors in the combination treatment group (13.88%) was 3.84-fold higher relative to the PBS control group (3.61%) (Fig. 5g–h), whereas no corresponding differences in CD4^+^ T cell infiltration were observed following combination treatment (Fig. 5g and Additional file 1: Fig. S8). Regulatory T cells (T_reg_s, CD4^+^Foxp3^+^) are important inhibitors of antitumor immunity, and the frequency of immunosuppressive T_reg_ cells was significantly reduced in distant tumors harvested from mice in the combination treatment group (18.55%) relative to the PBS control group (32.23%) (Fig. 5i–j), with a significant 42.44% decrease. As such, the systemic augmentation of cell-mediated immunity in mice subjected to combination SDT and immunotherapy treatment may account for the improved control of both primary and metastatic tumors.

### Combined SDT and anti-PD-1 treatment protects against tumor lung metastasis

**Fig. 6:**
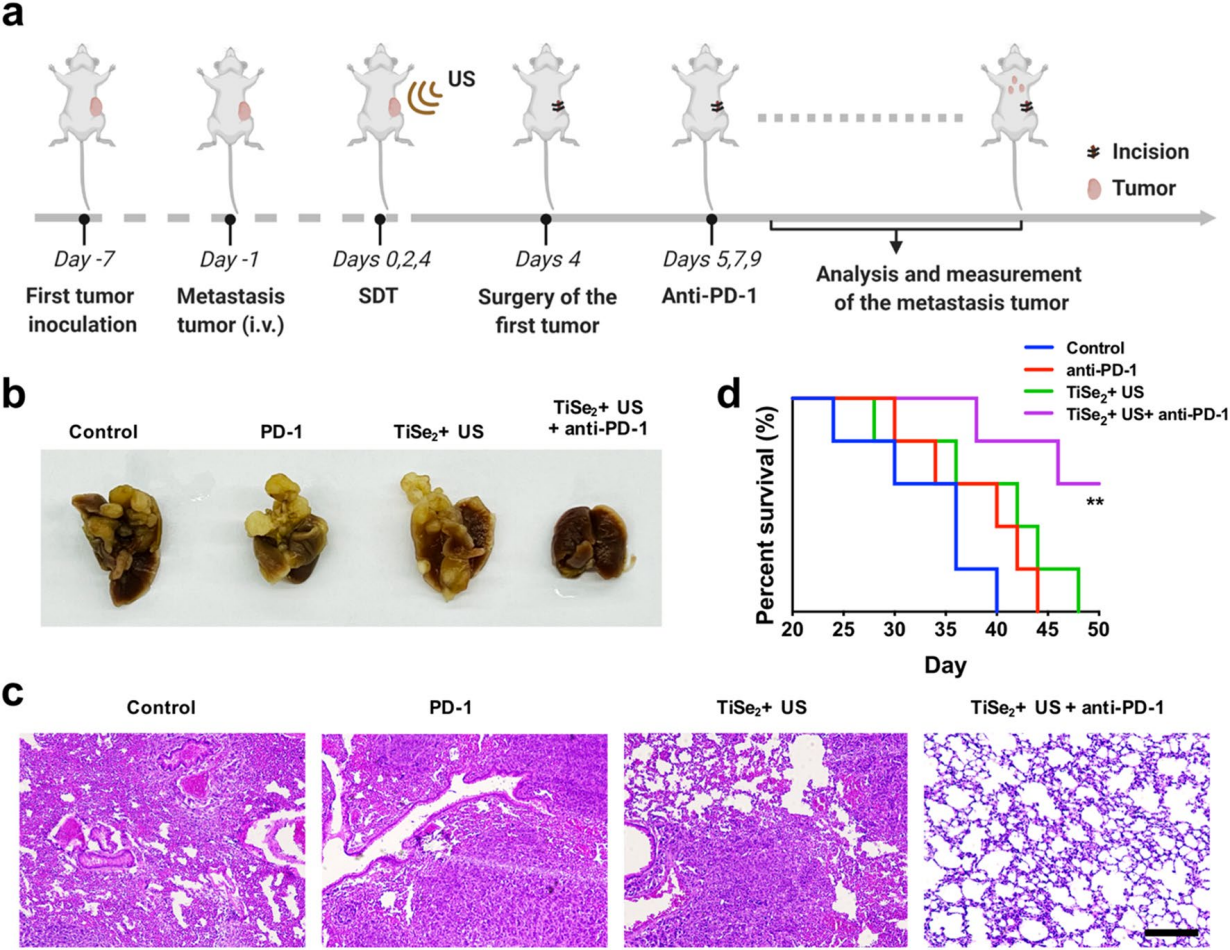
TiSe_2_-mediated SDT plus anti-PD-1 combination treatment suppresses tumor lung metastasis. **A** Schematic overview of the approach used to test the effects of TiSe_2_-mediated SDT and anti-PD-1 combination treatment on distant tumor metastasis. **b** Representative images of lungs bearing metastatic tumor nodules. **c** Representative H&E staining analysis of the lung metastasis. SDT, sonodynamic therapy; US, ultrasound. Scale bar: 200 µm. **d** Survival rates for mice in the indicated treatment groups bearing metastatic Pan02-luci tumors. Data were compared via Log-rank test. **P < 0.01

The above experimental approach was next extended to explore the ability of combination SDT and immunotherapy treatment to combat lung metastasis in an aggressive model of metastatic progression (Fig. 6a). Briefly, Panc02 cells were intravenously injected into unilateral subcutaneous Panc02 tumor-bearing mice to produce an artificial model of metastasis 6 days following initial tumor inoculation on the left flank. Mice were then subjected to three rounds of TiSe_2_-mediated SDT + anti-PD-1 treatment as above, and primary tumors were surgically excised on day 4. When lungs were excised from these mice at the end of the study period, significantly fewer lung nodules were visible in the lungs of mice from the SDT + anti-PD-1 group consistent with the ability of this combination treatment strategy to effectively prevent metastatic progression to the lungs (Fig. 6b). Following H&E staining, pathological analyses of these lungs similarly confirmed that mice which had undergone combination treatment harbored relatively few sporadic lung nodules, whereas mice in the PBS, anti-PD-1, and TiSe2 + US groups exhibited many tumor foci (Fig. 6c). Strikingly, 60% of mice in the TiSe_2_-mediated SDT + anti-PD-1 treatment group survived for 45 days following treatment, whereas no mice from any other treatment groups survived for this amount of time (Fig. 6d). Together, these results suggested that combining SDT and immunotherapeutic modalities was associated with robust anti-metastatic activity.

### In vivo biocompatibility of TiSe_2_ nanosheets

**Fig. 7:**
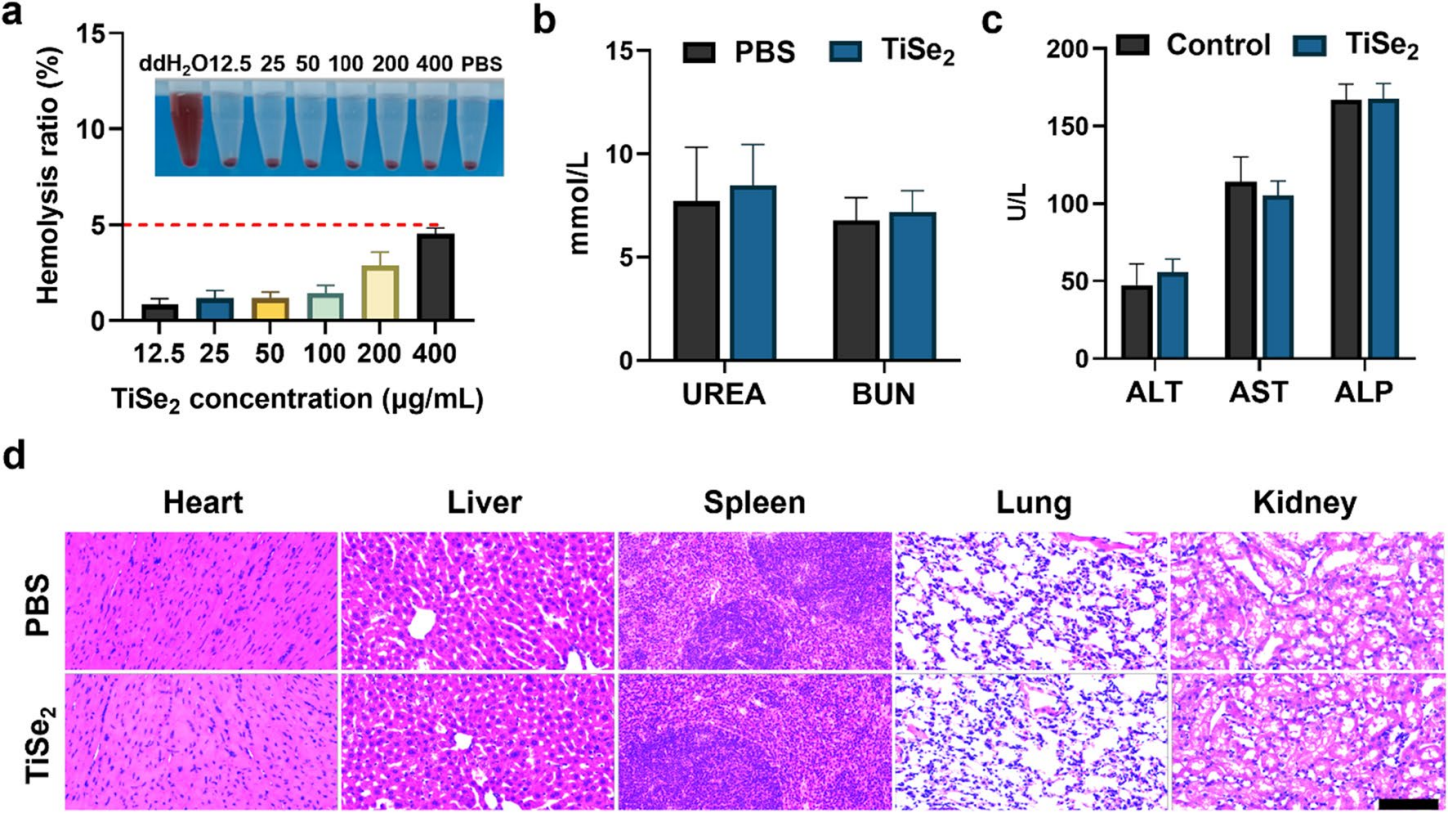
Analyses of the in vivo biosafety of prepared TiSe_2_ nanosheets. **a** A hemolysis assay was used to detect rates of RBC lysis in samples treated with ddH_2_O, PBS, or the indicated TiSe_2_ concentrations. **b**, **c** Murine renal (**b**) and hepatic (**c**) function was evaluated on day 14 following TiSe_2_ treatment. **d** H&E staining was used to examine major organs from mice including hearts, livers, lungs, spleens, and kidneys. Scale bar: 100 μm. Data are means ± SD (n = 3), and were compared via Student’s t-tests

Next, the safety of these synthesized TiSe_2_ nanosheets was analyzed by performing an in vitro hemolysis assay in which a range of TiSe_2_ concentrations were incubated with red blood cells from healthy mice. No significant hemolysis was evident in any group other than the positive control group in which cells were treated with water, thus confirming the excellent biocompatibility of these TiSe_2_ nanosheets (Fig. 7a). The toxicity of these TiSe_2_ nanosheets was further analyzed in vivo by administering them to healthy C57BL/6 mice. Treated animals did not exhibit any significant weight loss (Additional file 1: Fig. S9) or behavioral changes within the 14-day post-treatment period. Moreover, there was no evidence of any damage to renal (Fig. 7b) or hepatic function in these mice (Fig. 7c), nor were any pathological changes evident in major organs from mice in the TiSe_2_ nanosheet treatment groups relative to those of control mice following H&E staining (Fig. 7d). Overall, these data thus confirmed the good biosafety of these TiSe_2_ nanosheets, indicating that they are unlikely to induce any significant treatment-associated toxicity.

## Conclusions

In conclusion, TiSe_2_ nanosheets were herein synthesized and used as effective mediators of SDT that, when administered in combination with anti-PD-1 immune checkpoint blockade, were sufficient to effectively treat pancreatic cancer. When exposed to noninvasive US irradiation, these TiSe_2_ nanosheets can generate high levels of ROS under hypoxic or normoxic conditions, thus inducing the immunogenic death of tumor cells and thereby driving the maturation of DCs and the intratumoral infiltration of activated T cells. In addition to suppressing primary pancreatic tumor growth, this combination of TiSe_2_ nanosheet-mediated SDT and anti-PD-1 treatment also inhibited distant tumor growth and lung metastasis. Critically, this combination therapeutic modality was associated with the enhancement of systemic immune responses as evidenced by enhancement of DC maturation, cytokine secretion, as well as the increase of CD8^+^ T cells and the decrease of T_reg_ cells infiltrated in tumors of treated mice, conducive to the elimination of pancreatic tumors. Accordingly, these results highlight the promise of TiSe_2_ nanosheets as an immunotherapeutic platform for the effective treatment of pancreatic cancer.

## Supplementary Information

**Additional file 1: Figure S1. **Stability of the TiSe_2_ nanosheets incubated with PBS and 10% FBS-containing DMEM.** Figure S2.** Depletion of GSH *in vitro* after TiSe_2_-mediated SDT. **Figure S3.** Cell viability of Panc02 cells after US irradiation (1 MHz, 50% duty cycle, 1 min) for 24 h. **Figure S4.** The effect of the TiSe_2_+ US treatment on hTERT-HPNE cells and HUVEC cells. **Figure S5.** The biodistribution of Ti in major organs and tumors post intravenous injection with TiSe_2_ nanosheets for 8 h. **Figure S6.** The body weight changes during the TiSe_2_-mediated SDT + anti-PD-1 treatment. **Figure S7.** The temperature changes during the TiSe_2_-mediated SDT + anti-PD-1 treatment. **Figure S8. **Quantification of CD4^+^ T cells (CD45^+^CD4^+^) in mimic distant tumors. **Figure S9.** The body weight changes of healthy mice treated by PBS or TiSe_2_ nanosheets in 14 days.

## Abbreviations

TiSe_2_: Titanium diselenide
CTLA-4: Cytotoxic T lymphocyte antigen 4
PD-1: Programmed cell death protein 1
PD-L1: Programmed cell death ligand 1
TME: Tumor microenvironment
ICB: Immune checkpoint blockade
US: Ultrasound
SDT: Sonodynamic therapy
^1^O_2_: Singlet oxygen
ROS: Reactive oxygen species
ICD: Immunogenic cell death
DAMPs: Damage-associated molecular patterns
TAAs: Tumor-associated antigens
APCs: Antigen-presenting cells
DCs: Dendritic cells; NIR: near-infrared
TMDC: Transition metal dichalcogenides
PTT: Photothermal therapy
PDT: Photodynamic therapy
SOSG: Singlet oxygen sensor green
OH: Hydroxyl radical
APF: Aminophenyl fluorescein
ECL: Enhanced chemiluminescence
CLSM: Confocal laser scanning microscope
CRT: Calreticulin; HMGB1: high mobility group box 1
ATP: Adenosine triphosphate
CCK-8: Cell Counting Kit-8
TEM: Transmission electron microscopy
HR-TEM: High-resolution TEM
ICP-OES: Inductively plasma mass spectrometry
DLS: Dynamic light scattering
STEM-EDS: Energy dispersive X-ray spectroscopy-scanning TEM
XRD: X-ray diffraction
XPS: X-ray photoelectron spectroscopy
AFM: Atomic force microscopy
