# Supplementary material for: TiSe2-mediated sonodynamic and checkpoint blockade combined immunotherapy in hypoxic pancreatic cancer
Source: J Nanobiotechnology. 2022 Oct 15;20:453. doi: 10.1186/s12951-022-01659-4 (PMC9571469; doi:10.1186/s12951-022-01659-4)
Supplement: Supplementary file 1 — Additional file 1: Figure S1. Stability of the TiSe2 nanosheets incubated with PBS and 10% FBS-containing DMEM. Figure S2. Depletion of GSH in vitro after TiSe2-mediated SDT. Figure S3. Cell viability of Panc02 cells after US irradiation (1 MHz, 50% duty cycle, 1 min) for 24 h. Figure S4. The effect of the TiSe2+ US treatment on hTERT-HPNE cells and HUVEC cells. Figure S5. The biodistribution of Ti in major organs and tumors post intravenous injection with TiSe2 nanosheets for 8 h. Figure S6. The body weight changes during the TiSe2-mediated SDT + anti-PD-1 treatment. Figure S7. The temperature changes during the TiSe2-mediated SDT + anti-PD-1 treatment. Figure S8. Quantification of CD4+ T cells (CD45+CD4+) in mimic distant tumors. Figure S9. The body weight changes of healthy mice treated by PBS or TiSe2 nanosheets in 14 days. [file 12951_2022_1659_MOESM1_ESM.docx]

*Supporting Information*

**TiSe_2_-mediated sonodynamic and checkpoint blockade combined immunotherapy in hypoxic pancreatic cancer**

Libin Chen^1,2,5,#^, Wang Xue^3,4,#^, Jing Cao^3,4,#^, Shengmin Zhang^1^, Yiqing Zeng^3,4^, Ling Ma^1^, Xuechen Qian^1^, Qing Wen^3,4^, Yurong Hong^3,4^, Zhan Shi ^3,4,^*, Youfeng Xu^1,^*

*^1^ Department of Ultrasound in Medicine, Ningbo First Hospital, Ningbo 315010, P.R. China.*

*^2^* *Tongji University School of Medicine, Shanghai 200072, P. R. China.*

*^3^ Department of Ultrasound in Medicine, The Second Affiliated Hospital of Zhejiang University School of Medicine, Hangzhou 310009, P.R. China*

*^4^ Research Center of Ultrasound in Medicine and Biomedical Engineering, The Second Affiliated Hospital of Zhejiang University School of Medicine, Zhejiang University, Hangzhou, 310009, China.*

*^5^* *Department of Ultrasound in Medicine, Ningbo Ninth Hospital, Ningbo 315032, P.R. China.*

Fig. S1 Stability of the TiSe_2_ nanosheets incubated with PBS and 10% FBS-containing DMEM. Data are expressed as means ± SD (n= 5).


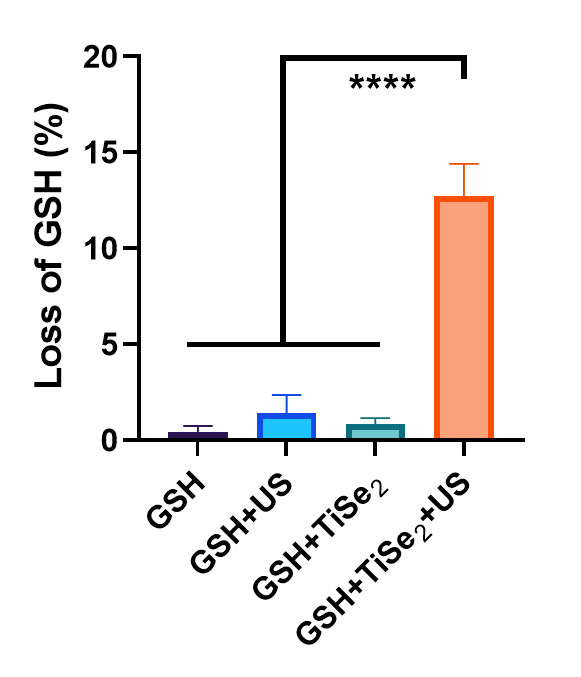


**Fig. S2** Depletion of GSH *in vitro* after TiSe_2_-mediated SDT. Data are expressed as means ± SD (n=3). Statistical significances were calculated by one-way ANOVA with a Bonferroni post-hoc test: ****P < 0.0001.

**Fig. S3** Cell viability of Panc02 cells after US irradiation (1 MHz, 50% duty cycle, 1 min) for 24 h. Data are expressed as means ± SD (n=4).

**Fig. S4** The effect of the TiSe_2_+ US treatment on hTERT-HPNE cells and HUVEC cells. Data are expressed as means ± SD (n= 5) and compared *via* one-way ANOVAs with Bonferroni post hoc testing.

**Fig. S5** The biodistribution of Ti in major organs and tumors post intravenous injection with TiSe_2_ nanosheets for 8 h. Data are expressed as means ± SD (n= 3).

**Fig. S6** The body weight changes during the TiSe_2_-mediated SDT + anti-PD-1 treatment. Data are expressed as means ± SD (n=5).

**Fig. S7** The temperature changes during the TiSe_2_-mediated SDT + anti-PD-1 treatment. Data are expressed as means ± SD (n=5).

**Fig. S8** Quantification of CD4^+^ T cells (CD45^+^CD4^+^) in mimic distant tumors. Data are expressed as means ± SD (n=5).

**Fig. S9** The body weight changes of healthy mice treated by PBS or TiSe_2_ nanosheets in 14 days. Data are expressed as means ± SD (n=3).
